# Antiproliferative and Molecular Mechanism of Eugenol-Induced Apoptosis in Cancer Cells

**DOI:** 10.3390/molecules17066290

**Published:** 2012-05-25

**Authors:** Saravana Kumar Jaganathan, Eko Supriyanto

**Affiliations:** 1 Department of Biomedical Engineering, PSNA college of Engineering and Technology, Kothandaraman Nagar, Dindigul 624622, Tamil Nadu, India; 2 Department of Clinical science and Engineering, University Technology Malaysia, Johor bahru 81310, Malaysia; Email: eko@utm.my

**Keywords:** eugenol, antiproliferative, cancer, chemoprevention

## Abstract

Phenolic phytochemicals are a broad class of nutraceuticals found in plants which have been extensively researched by scientists for their health-promoting potential. One such a compound which has been comprehensively used is eugenol (4-allyl-2-methoxyphenol), which is the active component of *Syzigium aromaticum* (cloves). Aromatic plants like nutmeg, basil, cinnamon and bay leaves also contain eugenol. Eugenol has a wide range of applications like perfumeries, flavorings, essential oils and in medicine as a local antiseptic and anesthetic. Increasing volumes of literature showed eugenol possesses antioxidant, antimutagenic, antigenotoxic, anti-inflammatory and anticancer properties. Molecular mechanism of eugenol-induced apoptosis in melanoma, skin tumors, osteosarcoma, leukemia, gastric and mast cells has been well documented. This review article will highlight the antiproliferative activity and molecular mechanism of the eugenol induced apoptosis against the cancer cells and animal models.

## 1. Introduction

Chemoprevention is defined as the administration of agents to prevent induction, to inhibit or to delay the progress of cancer [1], or as the inhibition or reversal of carcinogenesis at a premalignant stage [2]. Chemoprevention utilizes appropriate pharmacological agents [3,4] or of dietary agents, consumed in diverse forms like macronutrients, micronutrients, or nonnutritive phenolic phytochemicals [5,6,7]. Phenolic phytochemicals are one broad class of nutraceuticals found in plants which are extensively researched by scientists for their health-promoting potential.

Eugenol (4-allyl-2-methoxyphenol), one of these phytochemicals, is a biologically active phenolic component of *Syzigium aromaticum* (cloves). Eugenol has been used traditionally in Asian countries, mainly as a medicinal antiseptic, analgesic and antibacterial agent. Eugenol has been used as a flavoring agent in cosmetics and food products and also plays a role in dentistry as cavity filling cement [8].

Eugenol is said to possess various biological properties like antiviral, antioxidant, anti-inflammatory, *etc*. [8,9,10]. At low concentrations it usually acts as an antioxidant and anti-inflammatory agent, whereas at higher concentration act as a pro-oxidant causing increased generation of tissue-damaging free radicals [11,12]. It has been reported to possess antigenotoxic activity [13,14,15]. For instance, it suppressed the furylamide and aflatoxin B induced mutagencity in *Salmonella typhimurium* [13]. Eugenol also inhibited the DMBA-induced genotoxicity in MCF-7 cell line by modulating the detoxification enzymes like CYP1 and NAD (P) H: quinone oxidoreductase [14]. Moreover, oral feeding of eugenol along with *trans*-anethol also displayed antigenotoxic property against chemicals like cyclophosphamide, procarbazine and urethane [15].

Both the Food and Agriculture Organization (FAO) and World Health Organization (WHO) have allowed an acceptable daily intake of eugenol of 2.5 mg/kg body weight for humans [16]. Moreover, the U.S. Food and Drug Administration (FDA) has proclaimed eugenol as safe and it is considered non-carcinogenic and non-mutagenic. Recent studies depicted the anticancer activity of eugenol against various cancer cell lines and animal models. Additionally, the molecular mechanism of eugenol-induced apoptosis in melanoma, skin tumors, osteosarcoma, leukemia, gastric and mast cells has been well documented. The current review will highlight the antiproliferative activity and molecular mechanism of the eugenol-induced apoptosis. This article describes eugenol as a plausible candidate in cancer prevention.

## 2. Source, Chemistry and Structure of Eugenol

**Figure 1:**
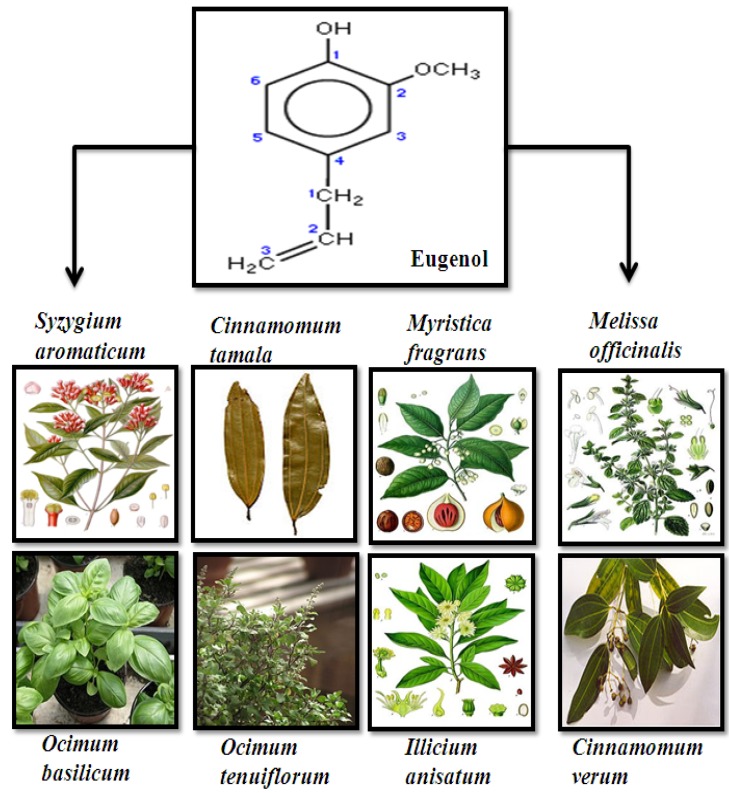
Structure and sources of eugenol.

The name eugenol originated from the scientific name of cloves: *Eugenia aromaticum* or *Eugenia caryophyllata* [17]. Essential oil extracted from the cloves contains almost 72–90% eugenol. Cloves are widely grown in Indonesia, Madagascar and also in other countries like India and Sri Lanka. Further, aromatic plants like *Cinnamomum tamala*, *Myristica fragrans*, *Melissa officinalis*, *Ocimum basilicum*, *Ocimum tenuiflorum*, *Illicium anisatum* and *Cinnamomum verum* also contain eugenol (Figure 1) [18,19,20]. Eugenol is a member of the allyl-benzene class of chemical compounds. It is an allyl chain-substituted guaiacol. Guaiacol is naturally occurring organic compound with the formula C_6_H_4_(OH) (OCH_3_). It appears as a clear to pale yellow oily liquid. Eugenol is generally well soluble in organic solvents and it is sparingly soluble in water.

## 3. Anti-Proliferative and Molecular Mechanism of Eugenol-Induced Apoptosis

Eugenol is reported to possess anticancer activity against various cancers. Additionally, the molecular mechanism of eugenol-induced apoptosis in melanoma, osteosarcoma, leukemia, gastric, skin tumors and mast cells has been well documented. The current review will delineate the antiproliferative activity and molecular mechanism of the eugenol-induced apoptosis in the above-mentioned cancer types.

### 3.1. Anti-Proliferative Mechanism of Eugenol against Melanoma Cells

Pisano *et al*. studied eugenol and six of its derivatives for its antiproliferative activity against primary melanoma cell lines [8]. Eugenol, isoeugenol monomers and their respective *O*-methylated forms (methyleugenol and methylisoeugenol) did not inhibit the melanoma cell proliferation at 100 µM. On the other hand, dimeric forms (biphenyls) of dehydrodieugenol decreased cell growth rate of about 40–60%. *O*, *O*'-dimethyldehydrodieugenol showed a growth inhibition of about 70–80% against the melanoma cells whereas the 6,6'-dibromodehydrodieugenol (S7) induced a fairly complete growth inhibition (nearly 100%) against the tested melanoma cell lines. The compound S7 is a chiral molecule and its enantiomeric form (S7-S) was found to be more potent compared to all others. Growth inhibitory activity of S7-S was specific to the melanoma and neuroblastoma cells. The IC_50_ values of S7-S against WM, GR, PNP, GILIN and LAN-5 cell lines were found to be 27, 23, 29, 19 and 16 µM respectively. S7-S treatment resulted in significant phosphatidylserine exposure in melanoma cells. Caspase activation was also detected after 50 µM of S7-S treatment. They concluded that the biphenyl eugenol-derivative enantiomer (*S*)-6,6'-dibromodehydrodieugenol (S7-S) has a potential to induce apoptosis in melanoma and neuroblastoma compared to other derivatives [8]. Kim *et al.* demonstrated eugenol-induced apoptosis in human melanoma cells [21]. Eugenol’s cytotoxic effects were observed in G361 cells in the range of 0.5 to 2 mM. At a concentration of 1 µM, it reduced the viability of G361 cells in a time-dependent fashion. Hoechst staining showed that eugenol induced a change in nuclear morphology. Eugenol-treated cells displayed condensed and fragmented nuclei compared to the typical round nuclei of the control. Gel electrophoresis indicated ladder pattern in the G361 cells after 1 mM eugenol treatment. Further Western blot analyses showed caspase-3 and caspase-6 activation. Moreover, caspase substrates like DFF45, PARP, lamin A were cleaved during eugenol-induced apoptosis [21]. In another study, Ghosh *et al.* explored eugenol and isoeugenol as an antiproliferative agent against malignant melanoma cells [22]. Eugenol was more potent in inhibiting melanoma cell lines compared to isoeugenol. Eugenol at a concentration of 0.5 µM inhibited 50% cell growth in Sbcl2 and WM3211 cell lines after 24 h. On the other hand, the WM98-1 and WM1205Lu cells needed twice as much time for 50% growth inhibition at this concentration of eugenol. Isoeugenol, an isomer of eugenol, could not inhibit the melanoma cell growth up to the concentration of 5 µM. Eugenol was also found to inhibit the colony formation of melanoma cell lines at 0.5 µM. Additionally, they tested the effect of eugenol against B16 melanoma xenograft. Eugenol caused significant tumor decrease (almost 40%) with a 19% increase in the median time to end point. Moreover, 50% of animals developed non-treatment related metastases while in the treatment group there was no sign of invasion or metastasis. TUNEL assay of the treatment groups proved eugenol induced apoptosis in the melanoma tumors. Cell cycle changes associated with the eugenol treatment of WM1205Lu cells were explored. It showed cells were blocked at S-phase (40%) accompanied by a decrease in the G1 phase cells with no significant change in the G2/M phase cells. Phase contrast microscopy and modified TUNEL assay of eugenol-treated WM1205Lu cells showed typical manifestations of apoptosis. Finally the cDNA array analysis showed that the E2F family of transcription factors have a role in the apoptosis induced by eugenol in melanoma cells. E2F1, E2F2, and E2F3 were all down-regulated by 2-fold or more with 1 µM eugenol treatment. E2F6, an E2F family member, was the only up-regulated factor after eugenol treatment. Since E2F1 was involved in cell cycle progression, they performed transient transfection assays and electrophoretic mobility shift assays to confirm that eugenol inhibits the transcriptional activity of E2F1. When E2F1 was overexpressed in the cells, it restored about 75% of proliferation ability in cultures, suggesting that eugenol could be developed as an E2F-targeted agent for melanoma treatment [22].

### 3.2. Antiproliferative Mechanism of Eugenol against Skin Tumors

Pal *et al.* evaluated the chemopreventive potential of eugenol in an experimental skin carcinogenesis mice model system [23]. Skin tumors were induced by topical application of DMBA croton oil in Swiss mice. Eugenol was orally administered 15 days prior to carcinogen treatment in order to ascertain the anticancer activity of eugenol. The number of mice that developed tumors was less in the eugenol-treated group (42%). Tumors were of a heterogeneous nature in control mice whereas in the eugenol-treated group they were suspected to be uniform papillomas. The mean height of the skin tumors was significantly higher in control group (1.789 cm) compared to the eugenol treated group (0.519 cm). Histopathological analysis showed that oral administration of eugenol could restrict the progression of carcinogenesis at the premalignant stage. *In-situ* proliferation assays suggested a higher proliferative index for the control group than for eugenol-treated animals. Further they studied the expression of some proliferation and apoptosis associated genes by RT-PCR and protein expression by Western blot. This lead to the conclusion that eugenol treatment resulted in the down-regulation of c-Myc, H-ras and Bcl_2_ expression along with up-regulation of p53, Bax and active caspase-3 expression in the skin lesions [23]. Another study initiated by Kaur *et al*. showed the protective effect of eugenol against cutaneous chemical carcinogenesis [24]. Skin tumors were initiated and promoted in mice by using 160 nm DMBA and 8.5 nm TPA for 28 weeks, respectively. All mice developed tumors by 13 week of promotion. Tumor multiplicity was 9.7 at the end of experiment for control, whereas eugenol pretreatment during the initiation and promotion stages caused reduction in tumor multiplicity to 5.6 and 2.8, respectively. Eugenol pretreatment delayed the tumor onset. PCNA and TUNEL analysis confirmed that eugenol induced apoptosis. Pretreatment with eugenol also resulted in the recovery of cellular GSH and various enzymes like activities of GR, CAT, GPX, GST and XO. Further eugenol augmented the p53 and p21 WAF1 levels in the DMBA-treated mice. The increased p53 expression resulted in the impediment of DMBA-induced neoplastic changes. Eugenol inhibited the ODC activity, iNOS, COX-2 expression and levels of pro-inflammatory cytokines (IL-6, TNF-α, PGE-2). It also had an inhibitory effect on the NF-kB, upstream signaling molecule, which regulates the expression of above-said genes. Moreover, they showed that eugenol could prevent the depletion of GSH and antioxidant enzymes caused by TPA. Hence they affirmed that eugenol could protect the chemically induced carcinogenesis by virtue of its antiproliferative, anti-inflammatory and antioxidant activities [24].

### 3.3. Antiproliferative Mechanism of Eugenol against Osteosarcoma Cells

Shin *et al.* studied the molecular mechanism of action of eugenol against osteosarcoma cells [25]. They showed eugenol could inhibit the HOS cell proliferation both in a dose and time-dependent manner. Eugenol at concentrations of 0.5, 1.0, 1.5, 2.0, 5.0 and 10.0 mM showed 91.7%, 83.1%, 56.6%, 25.3%, 13.2% and 8.4% survival rates after 24 h. At 2 mM concentration viability dropped to 84.8%, 53%, 25.3% and 5% of the control on treatment times 8, 16, 24 and 48 h, respectively. Hoechst staining showed untreated normal cells with homogeneous staining whereas apoptotic cells showed irregular staining of nuclei. Further DNA gel electrophoresis of 2 mM eugenol treated cells showed typical ladder pattern signifying apoptosis. Increased levels of p53, caspase 3 and cleaved PARP were evidenced in Western blot analyses. Cleavage of lamin A and cytosolic reduction of DFF-45 were also reported in the osteosarcoma cells after eugenol treatment [25].

### 3.4. Antiproliferative Mechanism of Eugenol against Leukemia Cells

Yoo *et al.* studied the anticancer mechanism of eugenol against human leukemia cells (HL-60) [26]. They initially estimated the IC_50_ values of eugenol against various cancer cell lines like U-937, HL-60, HepG2, 3LL Lewis, SNU-C5 which were found to be 39.4, 23.7, 118.6, 89.6 and 129.4 µM, respectively, after 48 h. In HL-60 cells, internucleosomal fragmentation of DNA was observed after 40 µM treatment for 4 h. However, the DNA fragmentation was completely blocked by pretreatment with the antioxidant *N*-acetyl-L-cysteine (NAC), suggesting ROS as a key player in the eugenol-induced apoptosis. ROS generation was detected using ROS-sensitive fluorometric probe, 2',7'-dichlorofluororescin (DCF), which showed increasing ROS generation after 40 µM treatment for 1 h. Further, eugenol depleted the intracellular GSH and protein thiols concentration both in a time and concentration-dependent manner. A significant difference in the cellular thiols was detected within 15 min of incubation using eugenol at concentrations of 20, 40 and 60 µM. There was a reduction in the mitochondrial membrane potential of the treated cells. Moreover, Western blot analyses of eugenol-treated cells indicated bax translocation, bcl_2_ reduction, cytochrome c release and caspase-9 and -3 activation [26]. Okada *et al.* [27] depicted the cytotoxicity and internucleosomal DNA fragmentation by 4-allyl-2-methoxyphenol (eugenol, EUG), 2-methoxy-4-methylphenol (MMP), 3,3'-dimethoxy-5,5'-di-2-propenyl-1,1'-biphenyl-2,2'-diol (bis-EUG) and 3,3'-di-methoxy-5,5'-dimethyl-1,1'-biphenyl-2,2'-diol (bis-MMP). Their results showed that bis-EUG was more potent in inducing DNA fragmentation and also more cytotoxic to HL-60 cells. RT-PCR analysis of the mRNAs for MnSOD and Cu/ZnSOD in HL-60 cells showed significant inhibition when exposed to 1 mM EUG for 1 h. Furthermore, inhibition of SOD mRNAs expression by EUG was strongly potentiated by the addition of 5 mM NAC or glutathione (GSH), whereas NAC or GSH alone did not affect the expression of SOD mRNAs. The cytotoxicity of EUG was significantly enhanced by high concentrations of NAC or GSH, which may be attributed to the inhibition of SOD mRNAs expression by the synergistic action of EUG and GSH or NAC. Northern blot analysis of lipopolysaccharide (LPS)-induced cyclooxygenase-2 (COX-2) gene expression in RAW 264.7 cells was performed. Bis-EUG, MMP and bis-MMP inhibited COX-2 gene expression at concentrations of 300, 500 and 500 µM, respectively. In contrast, no inhibitory effect of EUG was found over the wide concentration range of 10–500 µM, possibly as a result of the extensive mitochondrial dysfunction induced by this compound, which possesses potent pro-oxidative activity. Hence they concluded eugenol-related compounds, particularly bis-EUG, may act as nonsteroidal anti-inflammatory drug (NSAID)-like compounds [27].

### 3.5. Antiproliferative Mechanism of Eugenol against Gastric Cancer

Manikandan *et al.* investigated the eugenol-induced apoptosis in a rat model of gastric carcinogenesis induced by *N*-methyl-*N*'-nitro-*N*-nitrosoguanidine (MNNG) [28]. For this, they divided Wistar rats into four groups of six animals each. Group 1 received MNNG intragastric intubation of MNNG (150 mg/kg). Group 2 received MNNG as in Group 1, but received in addition intragastric administration of eugenol at 100 mg/kg. Group 3 received the eugenol alone at a concentration of 100 mg/kg, whereas Group 4 received only the basal diet (control). They reported that administering eugenol to MNNG treated animals (Group 2) significantly increased the mean final body weight compared to animals administered MNNG alone (Group 1). They also stated that eugenol decreased the tumor incidence to 16.66 per cent with a tumor burden of 14.78 mm^3^. They studied the role of NF-κB in MNNG-induced gastric carcinogenesis. Western, immunohistochemical and RT-PCR results showed that eugenol administration to MNNG treated animals significantly decreased the expression of NF-κB (p50 and p65), pIκBα and IKKβ and increased the expression of IκBα compared to MNNG treated animals. Further they studied the modulatory effects of eugenol against NF-κB target genes (cyclin D1, cyclin B, PCNA, p21, p53 and Gadd45). They reported that eugenol administration to MNNG treated animals significantly decreased the expression of cyclin D1, cyclin B and PCNA and increased the expression of p21, p53 and Gadd45 [28]. In their continuation study, they examined the markers for apoptosis, invasion and angiogenesis. They showed eugenol (100 mg/kg) administration decreased Bcl_2_ and Bcl-xL expression and increased the expression of Bax, Bid, Bad, Apaf-1, cytochrome C, caspase-9 and -3 and PARP cleavage compared to MNNG treated animals. Further they showed that in the eugenol-administered group decreased activities of MMPs (expression of MMP-2, MMP-9), VEGF and VEGFR1 and increased expression of TIMP-2 and RECK compared to Group 1 were observed [29]. In both studies where animals administered eugenol alone caused no significant changes compared to the untreated control (Group 4).

### 3.6. Antiproliferative Mechanism of Eugenol against Mast Cells

Park *et al.* described the apoptosis-inducing ability of eugenol against mast cells [30]. Mast cells are the granule-possessing secretory cells which modulate various immediate type allergies and inflammatory reactions. Moreover, potentials of various anti-allergic and anti-inflammatory drugs were demonstrated by their apoptosis-inducing ability in mast cells. In this article, they demonstrated the mechanism of eugenol-induced apoptosis in the mast cells. Eugenol reduced the cell viability of RBL-2H3 cells in a time and dose dependent manner. Half maximal inhibition (IC_50_) of eugenol in RBL-2H3 cells was found to be 700 µM. Similarly they also showed that eugenol could inhibit the growth of primary mast cells in a time and dose dependent manner. Apoptosis induction was confirmed by DNA ladder, activation of caspase-3 and the cleavage product of PARP 85 kda. Further they examined the role of mitochondria pathway in the eugenol-induced apoptosis. They found that level of Bax protein increased after 5 h and continued till 24 h. On the contrary they observed the level of Bcl_2_ decreased after 2 h of eugenol treatment. Concomitant reduction in the mitochondrial membrane potential was detected (using JC-1 stain) after 5 h treatment with eugenol. Further, cytochrome-c release from mitochondria to cytosol was evident in the immunofluorescent study, confirming the role of a mitochondrial pathway in the eugenol-induced apoptosis. Next, they examined the role of p53 in the eugenol-induced apoptosis of RBL-2H3. They reported eugenol increased the expression of phospho-ser 15-p53 without significant changes in the full length p53. Additionally phospho-ser 15-p53 was found to be initially translocated to the mitochondria within 2 h, which consequently reduced the mitochondrial membrane potential by interacting with Bcl_2_ and Bcl_xL_. p53-Specific antisense oligonucleotide prevented the apoptosis of RBL-2H3 cells due to eugenol. Hence they concluded that phospho-ser 15-p53 plays a pivotal role in eugenol-induced apoptosis of RBL-2H3 cells. Compound 48/80 is a polymer produced by the condensation of *N*-methyl-*p*-methoxy-phenethylamine with formaldehyde. It promotes histamine release and in biochemical research, compound 48/80 is used to promote mast cell degranulation [31]. In their *in vivo* studies, they showed that eugenol pretreatment prevented the degranulation of mast cells by compound 48/80 and also reduced the density of mesenteric mast cells. These results supported that survival of animals even after administration of the fatal dose of compound 48/80 might at least partly result from the decreased number of mast cells by eugenol pretreatment [30].

### 3.7. Antiproliferative Activity of Eugenol against Other Cancer Cells and Animal Model

Ghosh *et al.* probed the combinational effect of eugenol along with 2-methoxyestradiol (2-ME) against prostate cancer cells [32]. The IC_50_ values of 2-ME and eugenol in PC-3 cells were 1 µM and 82 µg/mL respectively. Similar levels of growth inhibition were achieved using lower concentrations of these agents in combination (0.5 µM 2-ME plus 41 µg/mL eugenol). They also showed 2-ME combined with eugenol inhibited LNCaP cell proliferation. Further cell cycle analysis displayed a significant increase of G_2_M phase by 4.6-fold when eugenol was combined with 2-ME compared to 2-ME alone. Phase contrast microscopy and annexin-V staining showed eugenol in combination with 2-ME induced significantly more apoptosis than alone. Bax and BCl_2_ also had their putative role in the synergistic combination of eugenol and 2-ME. Combination-induced apoptosis was not altered in PC-3 cells that over-express or lack Bcl_2_, but it correlated well with the loss of mitochondrial membrane potential. Hence they proclaimed that combining these two agents will reduce the effects of either 2-ME2 or eugenol alone [32]. Atsumi *et al.* synthesized dimer compounds from eugenol or isoeugenol and examined their cytotoxic activity against salivary gland tumor cell line (HSG) and normal human gingival fibroblast (HGF) [33]. The cytotoxic activity and the DNA synthesis inhibitory activity of these compounds were decreased in the order of dehydrodiisoeugenol > α-di-isoeugenol > isoeugenol > eugenol > bis-eugenol. They concluded that the significant cytotoxic activity of isoeugenol dimers was due to an interaction with cell membranes via the lipophilic radical [33]. Carrasco *et al.* [34] probed eugenol and its derivatives against two cancer cells, namely DU-145 (androgen-insensitive prostate cancer cells) and KB (oral squamous carcinoma cells). In the examined cancer cells, eugenol and its derivatives showed cell-growth inhibition activity. Further they demonstrated that eugenol derivatives like 5-allyl-3-nitrobenzene-1,2-diol and 4-allyl-2-methoxy-5-nitrophenyl acetate were significantly (*p* < 0.001) more active than eugenol, with IC_50_ values of 19.02 × 10^−6^ and 21.5 × 10^−6^ mol L^−^^1^ in DU-145 cells, respectively, and in KB cells it was found to be 18.11 × 10^−6^ and 21.26 × 10^−6^ mol L^−1^ respectively. Hence they suggested that the presence of nitro and hydroxyl groups in these derivatives may have a putative role in the activity of these compounds [34]. In a recent study by Jaganathan *et al.* they proposed the anticancer activity of eugenol against Ehrlich ascites and solid carcinoma [35]. They showed that eugenol at a dose of 100mg/kg i/p was able to inhibit the growth of Ehrlich ascites by 28.88%. In case of solid carcinoma, eugenol (100mg/kg; i/p) showed 24.35% tumor growth inhibition. Further in their follow up study they tested the apoptotic effect of eugenol against colon cancer cells. They elicited that eugenol transduced the apoptotic signal via depletion of non-protein thiols, decreasing the mitochondrial membrane potential and consequently increasing the ROS generation. Augmented ROS generation resulted in the DNA fragmentation, a hallmark of apoptosis, in the eugenol-treated colon cancer cells. Further, ROS generation was accompanied with increasing p53 activation and PARP cleavage. Finally, caspase-3 resulted in the enhanced apoptosis of colon cancer cells [36]. In another independent research, tumors (papillomas) produced by the application of 7,12-dimethyl benz(a)anthracene as an initiator and croton oil as promoter in mice were considerably inhibited by the prior application of eugenol [37]. Recent study performed by Hussain *et al*. [38] investigated the effect of combination of eugenol and sulforaphane (SFN) against human cervical cancer cells. SFN and eugenol in combination treatment resulted in the differential effects likely an antagonistic effect at lower and synergistic at higher sub-lethal doses as revealed in cell cytotoxicity and apoptosis induction. Moreover when gemcitabine was used in conjunction with the low- and high-dose combinations, it showed no significant cell death at lower doses suggesting that cell cytotoxicity is proportional to gemcitabine alone, whereas at higher sublethal doses of SFN and eugenol, they were found to act in a synergistic manner with gemcitabine [38], so they extended their study to see whether eugenol enhances the chemotherapeutic potential of gemcitabine. Eugenol displayed dose-dependent selective cytotoxicity toward HeLa cells in comparison to normal cells, indicating its safe cytotoxicity profile. The value of combination index was <1 indicating strong synergistic interaction. Hence they concluded that the combination of eugenol may enhance the efficacy of gemcitabine at lower doses and minimize the toxicity on normal cells [39]. Vidhya *et al*. investigated eugenol for its anticancer effect in human breast cancer cells (MCF-7) [40]. Eugenol inhibited the MCF-7 cell growth both in dose and time dependent manner. Eugenol-treated cells showed cell shrinkage, membrane blebbing and apoptotic body formation. Further, eugenol treatment also depleted the level of intracellular glutathione and increased the level of lipid peroxidation.

## 4. Conclusions

Chemoprevention utilizes appropriate pharmacological agents [3,4] or dietary agents, consumed in diverse forms like macronutrients, micronutrients, or nonnutritive phenolic phytochemicals [5,6,7]. Eugenol (4-allyl-2-methoxyphenol), one of these phytochemicals, is a biologically active phenolic component of *Syzigium aromaticum* (cloves). Eugenol is said to be possess various biological properties such as antioxidant, antimutagenic and anti-inflammatory effects, *etc*. Recent studies showed the anticancer activity of eugenol against various cancer cell lines and animal models. A summary of antiproliferative effects and their molecular mechanism are listed in Table 1.

**Table 1:** Antiproliferative and molecular mechanism of eugenol-induced apoptosis.

Tested Compound	Cancer Type	Observations/Results	Reference
Eugenol/Isoeugenol	Melanoma	► no significant activity at 100 µM	[8]
Dehydrodieugenol	Melanoma	► 40–60% growth inhibition	[8]
*O*, *O*'-Dimethyl-dehydrodieugenol (S7)	Melanoma	► 70–80% growth inhibition	[8]
6,6'-Dibromo-dehydrodieugenol	Melanoma	► nearly 100% growth inhibition	[8]
		► The IC_50_ of S7-S against WM, GR, PNP, GILIN, LAN-5 cell lines were found to be 27, 23, 29, 19 and 16 µM respectively	
		► 50 µM of S7-S exposure for 24 h resulted in DNA fragmentation as detected by TUNEL assay	
Eugenol	Melanoma	► cytotoxic effect was observed in G361 cells in the range of 0.5 to 2 mM	[21]
		► caspase-3 and caspase-6 activation	
		► caspase’s substrate like DFF45, PARP, Lamin A were cleaved	
Eugenol	Melanoma	► concentration of 0.5 µM inhibited 50% cell growth in Sbcl2 and WM3211 after 24 h	[22]
		► in B16 melanoma Xenograft, it caused significant tumor decrease (almost 40%)	
		► TUNEL assay of WM1205Lu cells confirmed apoptosis	
		► E2F family of transcription factors have a role in the apoptosis	
Eugenol	Osteosarcoma	► inhibited the HOS cell proliferation both in dose and time-dependent manner	[25]
		► increased levels of p53, caspase 3 and cleaved PARP	
		► cleavage of lamin A and cytosolic reduction of DFF-45	
Eugenol	Leukemia	► concentration of 23.7 µM inhibited 50% cell growth in HL-60	[26]
		► increased ROS generation and GSH depletion	
		► increased bax translocation, bcl_2_ reduction, cytochrome c release and caspase-9 and -3 activation	
Eugenol	Gastric cancer	► decreased the expression of NF-κB (p50 and p65), pIκBα and IKKβ and increased the expression of IκBα	[28,29]
		► decreased the expression of cyclin D1, cyclin B, and PCNA and increased the expression of p21, p53 and Gadd45	
		► decreased Bcl_2_ and Bcl-xL expression, and increased the expression of Bax, Bid, Bad, Apaf-1, cytochrome C, and caspase-9, and -3 and PARP	
		► decreased the activities of MMPS (expression of MMP-2, MMP-9), VEGF and VEGFR1 and increased the expression of TIMP-2 and RECK	
Eugenol	Skin tumor	► number of mice that developed tumors was less in the eugenol treated group (42%)	[23]
		► resulted in the down-regulation of c-Myc, H-ras and Bcl_2_ expression along with up-regulation of p53, Bax and active caspase-3 expression in the skin lesions	
Eugenol	Skin tumor	► PCNA and TUNEL analysis confirmed apoptosis	[24]
		► recovery of cellular GSH and various enzymes like activities of GR, CAT, GPX, GST, and XO	
		► increased p53 and p21 WAF1 levels	
		► inhibition of ODC activity, iNOS, COX-2 expression	
		► decreased levels of pro-inflammatory cytokines (IL-6, TNF-α, PGE-2) and NF-κB	
Eugenol	Mast cells	► half maximal inhibition (IC_50_) of Eugenol in RBL-2H3 cells was found to be 700 µM	[30]
		► apoptosis induction was confirmed by DNA ladder, activation of caspase-3 and the cleavage product of PARP 85 kda	
		► phospho-ser 15-p53 plays a pivotal role in eugenol-induced apoptosis of RBL-2H3 cells	
Eugenol	Prostate cancer	► combinational effect of eugenol along with 2-methoxyestradiol (2-ME) against prostate cancer cells	[32]
		► cell cycle analysis displayed significant increase of G_2_M phase by 4.6-fold, when eugenol was combined with 2-ME	
		► Bax and Bcl_2_ had a role in the synergistic combination	

Pisano *et al*. studied eugenol and six of its derivatives for their antiproliferative activity against primary melanoma cell lines [8]. They concluded that the biphenyl eugenol-derivative enantiomer (*S*)-6,6'-dibromo-dehydrodieugenol (S7-S) has a potential to induce apoptosis in melanoma and neuroblastoma compared to other derivatives. Eugenol treatment resulted in the apoptosis of G361 cells with the possible association of caspases 3 and 6. Moreover caspase substrates like DFF45, PARP, lamin A were cleaved during eugenol-induced apoptosis in G361 cells. Ghosh *et al.* [22] suggesting that eugenol could be developed as an E2F-targeted agent for melanoma treatment. Osteosarcoma cell (HOS) proliferation was inhibited by eugenol both in dose and time-dependent manner. Increased levels of caspase-3, p53 and PARP cleavage accompanied the eugenol-induced apoptosis in HOS cells [25]. Yoo *et al*. studied the anticancer mechanism of eugenol against human leukemia cells (HL-60) [26]. Increased ROS generation, depletion of GSH and protein thiols, and a fall in the mitochondrial membrane potential and activation of caspase 3 were observed in the apoptosis of HL-60 cells.

Manikandan *et al*.investigated the eugenol-induced apoptosis in a rat model of gastric carcinogenesis induced by *N*-methyl-*N*-nitro-*N*-nitrosoguanidine (MNNG). They provided evidence for the protective effect of eugenol against MNNG-induced gastric carcinogenesis. They attributed this to eugenol’s ability to inhibit NF-κB activation. They also showed eugenol-induced apoptosis via the mitochondrial pathway by modulating the Bcl_2_ family proteins, angiogenesis and invasion in their investigated models [28,29]. Pal *et al*. evaluated the chemopreventive potential of eugenol in the experimental skin carcinogenesis mice model system [23]. They concluded that eugenol treatment resulted in the down-regulation of c-Myc, H-ras and Bcl_2_ expression along with up-regulation of p53, Bax and active caspase-3 expression in the skin lesions. Another study initiated by Kaur *et al*. [24] showed the protective effect of eugenol against cutaneous chemical carcinogenesis. Park *et al*. [30]described the pivotal role of phospho-ser 15-p53 in the apoptosis induced by eugenol against the mast cells.

Our review summarized the antiproliferative and molecular mechanism of eugenol-induced apoptosis in various cancer cell lines and animal models. Eugenol has evolved as a promising candidate both in *in vivo* and *in vitro* studies. However before promoting this candidate for clinical trials, further in-depth information of this agent in laboratory setups has to be generated.
